# Impact of Solid Fuel Use on Household Air Pollution and Respiratory Health in Two Low-Income Communities in Mpumalanga, South Africa

**DOI:** 10.5334/aogh.4923

**Published:** 2025-10-08

**Authors:** Bianca Wernecke, Kristy Langerman, Angela Mathee, Nada Abdelatif, Marcus A. Howard, Nkosana Jafta, Christiaan Pauw, Shumani Phaswana, Kareshma Asharam, Ishen Seocharan, Hendrik Smith, Rajen N. Naidoo, Natasha Naidoo, Caradee Y. Wright

**Affiliations:** 1 Department of Geography, Geoinformatics and Meteorology, University of Pretoria, Pretoria, South Africa; 2 Climate Change and Health Research Programme, Environment and Health Research Unit, South African Medical Research Council, Pretoria, South Africa; 3 Department of Geography, Environmental Management and Energy Studies, University of Johannesburg, Johannesburg, South Africa; 4 Environment and Health Research Unit, South African Medical Research Council, Johannesburg, South Africa; 5 Department of Environmental Health, Faculty of Health Sciences, University of Johannesburg, Doornfontein, Johannesburg, South Africa; 6 Biostatistics Research Unit, South African Medical Research Council, Durban 4091, South Africa; 7 Nova Institute, Pretoria, South Africa; 8 Discipline of Occupational and Environmental Health, School of Nursing and Public Health, College of Health Sciences, University of KwaZulu-Natal, Durban, South Africa

**Keywords:** air quality, allergies, coal use, household air pollution, indoor air quality, low-income communities, particulate matter, PM_2‧5_, respiratory health, solid fuels

## Abstract

*Introduction:* Household air pollution from domestic solid fuel use remains a global public health concern, particularly in low-income communities. This study assessed associations between household fuel use, indoor air pollution, and respiratory health outcomes in two Mpumalanga communities in South Africa.

*Methods:* A cross-sectional study was conducted in KwaZamokuhle and eMzinoni between July 2019 and February 2020. Indoor PM_2‧5_ concentrations were measured using Airmetrics MiniVol samplers and TSI DustTrak II monitors. We carried out household surveys, lung function tests and allergen sensitivity testing and performed multivariable logistic regression to assess associations between indoor pollutant exposure and respiratory health outcomes.

*Results:* Indoor and ambient PM_2‧5_ concentrations in KwaZamokuhle were more than twice as high as those in eMzinoni, exceeding both national standards and WHO Air Quality Guidelines. Coal use for heating was more prevalent in KwaZamokuhle and appeared directly related to elevated PM_2‧5_ levels. Approximately 9% of participants exhibited signs of obstructive airway disease, and 25% had positive results for allergen sensitisation. Although the associations between PM_2‧5_ levels, solid fuel use and measured respiratory outcomes did not reach statistical significance, consistent trends in the expected direction were observed, suggesting a potential relationship that warrants longitudinal studies with larger sample sizes.

*Conclusion:* These findings suggest complex, possibly nonlinear relationships between indoor air pollution and respiratory health effects. The study underscores the urgent need for a greater use of clean energy alternatives and increased public awareness about the risks of household air pollution in low-income South African communities.

## Introduction

Household air pollution (HAP) causes ~3.2 million premature deaths globally every year, primarily in low- and middle-income countries (LMICs), where polluting fuels and low-resource biomass cookstoves are commonly used in homes for cooking and heating [1, 2]. The most significant contributor to HAP is smoke from cooking fires [2]. Women and children are most affected as they typically spend more time indoors [2].

Despite a slight decline in HAP-related morbidities, multiple studies show elevated risks of acute respiratory infections in children and adults in African countries from cooking with polluting fuels [3]. In studies comparing dirty fuels with clean fuels like electricity, the adjusted odds ratios (aOR) are: 1.44 (95% CI: 1.21–1.92) for polluting fuels overall, 1.10 (95% CI: 0.79–1.53) for wood, 1.54 (95% CI: 1.02–2.33) for animal dung and 4.35 (95% CI: 1.63–11.6) for charcoal [3–6]. According to a recent national burden of disease study in South Africa, LRIs and COPD are the first and fifth topmost causes of death attributable to HAP exposure, respectively [7]. Additional population-based studies are needed to generate robust estimates of the attributable risk of HAP for asthma and other respiratory conditions.

Particulate matter with a diameter of 2.5 µm or smaller (PM_2.5_) is associated with the increased prevalence of allergic respiratory disease, including allergic rhinitis and allergic asthma, as it enhances allergic inflammatory responses [8, 9]. Air pollution from solid fuel combustion and other microbial indoor pollutants like dust, pollen, moulds and pet dander can interact to cause increased Immunoglobulin E (IgE), an antibody produced in response to allergen exposure [10]. The prevalence of allergic diseases in LMICs is increasing [11], including in countries like South Africa [12].

Most studies examining the effects of HAP on asthma and other respiratory symptoms have focused on children, particularly within school environments. A multinational study was conducted across six cities in sub-Saharan Africa, including Blantyre (Malawi), Durban (South Africa), Harare (Zimbabwe), Kumasi (Ghana), Lagos (Nigeria) and Moshi (Tanzania) [13]. The study highlighted the critical importance of household air quality in asthma control [13]. One of the key adaptation interventions identified was the increased use of clean fuels for cooking and lighting in homes, which would contribute to improved respiratory health outcomes [13]. A few studies in South Africa have found significant associations between ambient air pollution and aggravated asthma symptoms in children [14–16].

A recent study showed household PM_2.5_ concentrations in Soweto (urban) and Agincourt (rural) areas frequently and significantly exceeded WHO Air Quality Guidelines, especially during winter [17]. The hazard quotients exceeded 1 in nearly half of rural households and remained consistently >1 in urban areas across both seasons, indicating significant health risks [17]. Indoor pollutant levels were frequently higher than outdoor concentrations, highlighting the risks from indoor solid fuel burning [17].

Despite the pressing nature of South Africa’s respiratory illness health burden, there are insufficient studies providing measured exposure and health outcome data [18, 19]. Recognising these shortcomings, the aim of this study was to understand the relationships between fuel use patterns, PM_2.5_ exposure and associated respiratory health outcomes among households in KwaZamokuhle and eMzinoni, two low-income communities located in Mpumalanga Province, South Africa. In the two communities, the study objectives were: (1) to determine the primary energy carriers used for cooking, heating and lighting; (2) to quantify indoor and ambient PM_2.5_ concentrations; and (3) to assess the relationship between air pollutants and the occurrence of asthma and respiratory symptoms among women responsible for cooking in low-income households. This facilitated a comparative analysis of risk factors associated with domestic fuel consumption and related air pollution considering their impact on pulmonary function and allergen sensitivity in two low-income communities.

## Materials and Methods

### Study area

**Figure 1:**
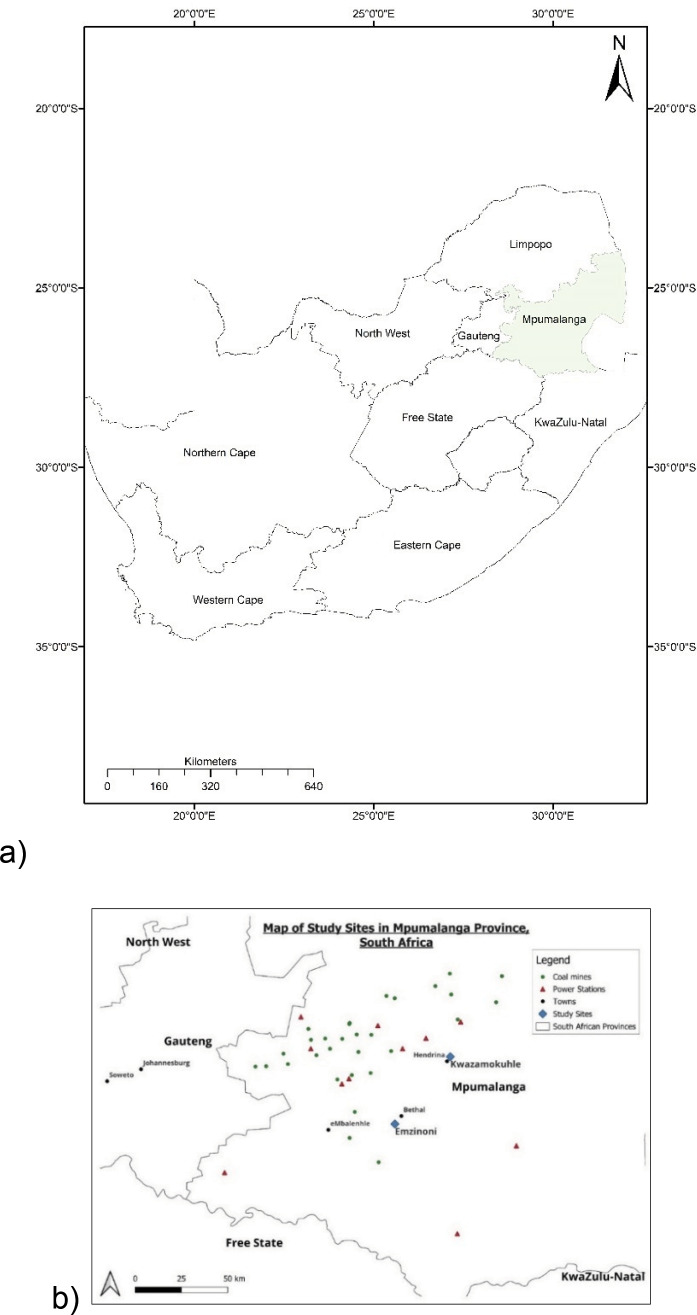
Location of **(a)** Mpumalanga Province in South Africa and **(b)** KwaZamokuhle and eMzinoni in Mpumalanga Province.

The study took place in the communities of KwaZamokuhle and eMzinoni in Mpumalanga Province, South Africa, between July 2019 and February 2020 (Figure 1). A large proportion of households use coal for cooking and heating despite having access to electricity [20]. In the study area, coal is readily available and cheap given the community’s proximity to coal mines [20].

### Community, household and participant selection

The study originally aimed to enrol 1000 participants, 500 per site and sites selected as part of a large, non-randomised control trial aimed at identifying the health impacts of HAP reduction interventions in low-income communities. The towns were selected based on similar demographics, geography and proximity to industrial air pollution sources such as coal mines and power stations. The study was prematurely halted after baseline data collection phase due to the COVID-19 pandemic. The cross-sectional data for these two communities were applied in the analyses here.

**Figure 2:**
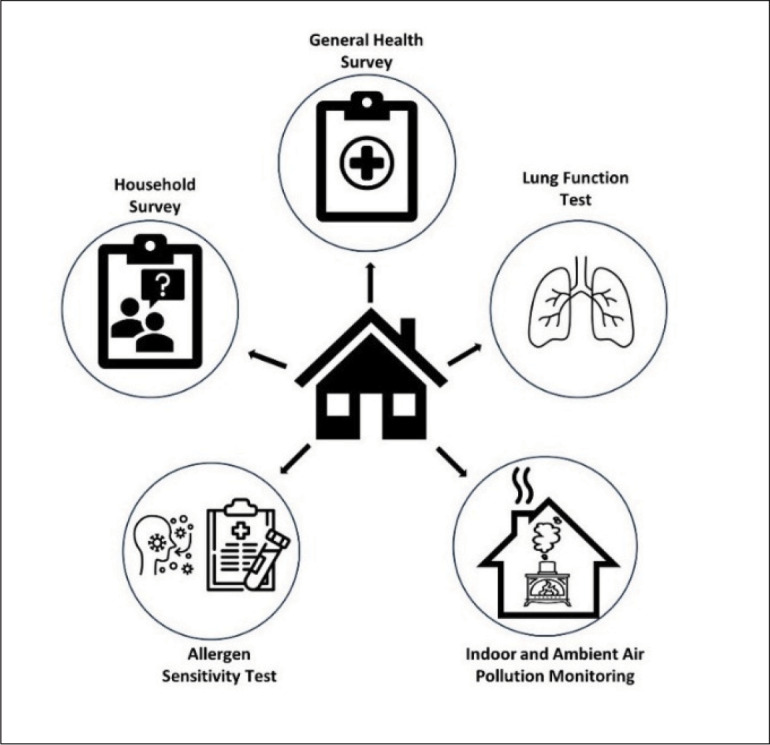
Overview of the surveys, tests and data monitoring conducted in and around participating households.

Households living in formal houses (defined as permanent residential structures with access to municipal services) across both communities were recruited. Dwelling selection took place according to a matched pair strategy. At least 20 cluster-matched pairs of household groupings were selected based on local criteria, e.g., proximity to a school, clinic, busy road or police station. This design allowed for analysis to be done at a household level adjusting for confounding variables and reducing variability within the data. Within each of these clusters, households were randomly selected. Depending on the consent response of the study participants and other external limiting factors (e.g., the unavailability of a household member during a survey or the onset of lockdown during COVID-19) the sample sizes for the various surveys differed (Figure 2).

### Household and general health survey

A household questionnaire (Supplementary Table S1) was administered to all participating households after having been field validated and piloted. Data on demography (e.g., age, education and employment status), socio-economic status (e.g., monthly income), housing conditions (e.g., water source), fuel use patterns (e.g., main fuel used for heating and cooking) and smoking practices (e.g., whether a household member smokes) were collected. The household questionnaire was completed by the woman in the household who was most frequently tasked with cooking.

Fieldworkers from the local communities were recruited and trained to administer the questionnaires. Responses were logged using mobile tablets and mobile data capture software called RedCap [21]. Pilot study results were not included in the final dataset. A random sample (~10%) of interviews was selected for quality control re-interviews via telephone. A selection of questions was re-asked of participants to confirm that the original answers were true.

### Indoor air pollution monitoring

Airborne PM_2.5_ was sampled inside a subset (*n* = 113) of the households across both communities using battery-operated Airmetrics MiniVol samplers with interchangeable impactors and with continuous TSI DustTrak monitors for 24-hour periods in each house (DustTrak II Aerosol Monitor 8530) [22]. Monitoring took place during winter and summer between July 2019 and February 2020. The subset was necessary due to the number of available instruments and budget constraints.

Concentrations measured by the MiniVols were determined gravimetrically. A microgram scale was used to weigh the filters pre- and post-exposure. PM_2.5_ concentrations in the households were calculated using the change in filter weight and the volume sampled during the collection of PM. The weight of unexposed filters, as well as adjusted flow rates, was taken into consideration. Gravimetric analyses were undertaken at the laboratories of the University of KwaZulu-Natal. The cleaning, leak checks and single-point flow rate checks were conducted for the MiniVols as per the manufacturer’s specifications.

The DustTrak instruments were factory calibrated before being deployed in the field and then zero calibrated and flow checked as per the manufacturer’s directions. Concentrations were expressed in micrograms per cubic meter (µg/m^3^) per 24-hour period on weekdays.

### Ambient air quality monitoring

Ambient air quality data were sourced from government-owned, centrally located monitoring stations in each community, and each household was assigned ambient air quality data from the station nearest to them. Hourly averages were downloaded in µg/m^3^ for the same period for which indoor monitoring took place. These concentrations were then averaged per day for direct comparison with the indoor measurements.

### Lung function test

Participants from consenting households took part in lung function testing using the Easy One (NDD Medizintachnik AG) Spirometry device [23]. The lung function tests were administered by a qualified pulmonary technologist using the American Thoracic Society guidelines for conducting spirometry. Spirometry was performed in a sitting position without nose clips, and participants were asked to blow into the device. A clean mouthpiece was used for every individual. Indices of primary interest included forced vital capacity (FVC) and forced expiratory volume in one second (FEV1) both measured in litres. The FEV1/FVC ratio was used in the diagnosis of obstructive airways disease. The ratio represents the proportion of a person’s vital capacity. In obstructive airways disease, the FEV1 is reduced due to an obstruction of air escaping from the lungs. This could, for example, be due to inflammation in the airways from exposure to air pollution [24]. The diagnosis of obstructive airways disease is made when the FEV1/FVC ratio is less than 0.7 [25].

### Allergen sensitivity test

An allergy screening test for inhalant allergens was conducted. The immunological status of participants was assessed by means of a blood IgE test (a non-specific test for allergy to allergens). A qualified phlebotomist took blood specimens. The blood was transported using standard operating procedures for analytic laboratories from the test site to the laboratory service provider. A positive result indicated that the participant’s immune system has produced IgE antibodies in response to exposure to allergens [26]. The test is considered positive if the total IgE antibody is equal to or above 0.35 KU/L [27]. This sensitisation is the first step to developing allergies but does not automatically mean that the person will show clinical allergy symptoms [26].

## Research Ethics

Each participant was required to give informed consent to partake in the study for each survey. Research Ethics Clearance was granted by the South African Medical Research Council Research Ethics Committee (Study ID EC006-4/2019). Trained individuals conducted all medical tests and participants were informed of their test results.

## Data and Statistical Analysis

### A comparison of respiratory health and pollutant exposure across communities

A comparison was undertaken to assess the impact of differing levels of residential fuel use and PM exposure on respiratory health in the two communities. Data were analysed using Stata 18 [28] and were summarised descriptively using frequencies for categorical variables and medians and interquartile ranges for the continuous variables as these were non-normally distributed. The prevalence of health outcomes was evaluated per community. Respiratory health outcomes were categorised into self-reported and objectively measured health outcomes. Cross-tabulations were conducted to quantitatively analyse the relationship between exposure variables and measured health outcomes. Ambient and indoor air pollution daily means were calculated and compared to the National Ambient Air Quality Standards (NAAQS) [29].

### Tests for differences in air pollution levels and health outcomes between communities

The Mann–Whitney *U* test was used to study the differences between indoor and ambient air pollutant concentrations and the respiratory health of participants between the two communities. The chi-square test and Fisher’s exact test were used to evaluate associations between categorical variables like fuel use for larger and smaller sample sizes, respectively. *p*-values were considered statistically significant if they were less than 0.05.

### Regression analysis to assess associations between exposure and health outcomes

Multiple logistic regression analyses were performed using the pooled, binary lung function and allergen sensitivity test results as health outcomes (i.e., the presence or absence of obstructive airways disease or the positive or negative allergen sensitivity result). The variable ‘town’ was used as a surrogate for exposure. Based on the results of the fuel use and air quality assessments, eMzinoni was treated as the reference category. Crude and adjusted odds ratios (OR) with 95% confidence intervals (CI) were calculated to examine the relationship between measured health outcomes and a range of independent variables linked to fuel use (as HAP proxies) as well as identified confounders. Confounding variables were selected based on previous findings as well as univariate analyses [30]. We also made sure that these variables were not highly correlated with each other and identified the best statistical model using established criteria, namely the Akaike Information Criterion (AIC) and Bayesian Information Criterion (BIC). In the multivariable models, we adjusted for employment status, monthly income, education and age, with eMzinoni specified as the reference site.

### Sequential modelling of exposure and health outcome associations

Three models were run separately for each exposure and health outcome combination. The first was the baseline bivariate analysis, or the unadjusted model (called ‘baseline’). The second model adjusted for the living conditions of the participants that might have influenced exposure levels (e.g., how long they lived in their community and whether they had a ceiling or not) (‘living conditions’). Finally, the third model adjusted for socio-economic factors, including employment status, education level, age and monthly income (‘socio-economic factors’).

### Justification for exclusion of PM_2.5_ measurements in regression analyses

Measured indoor PM_2.5_ concentrations were not used as exposure variables in the bivariate or multivariate regression analyses as one daily average PM_2.5_ reading per dwelling was not considered sufficiently representative of the highly variable HAP levels to which a participant is exposed. As ambient PM_2.5_ measurements represent concentrations measured at one stationary location, they were not considered an adequate exposure proxy. Fuels used for heating and cooking, as well as stove types used, were grouped into ‘non-electric’ and ‘electric’ categories. Electric categories were treated as reference categories.

## Results

### Sample description

**Figure 3:**
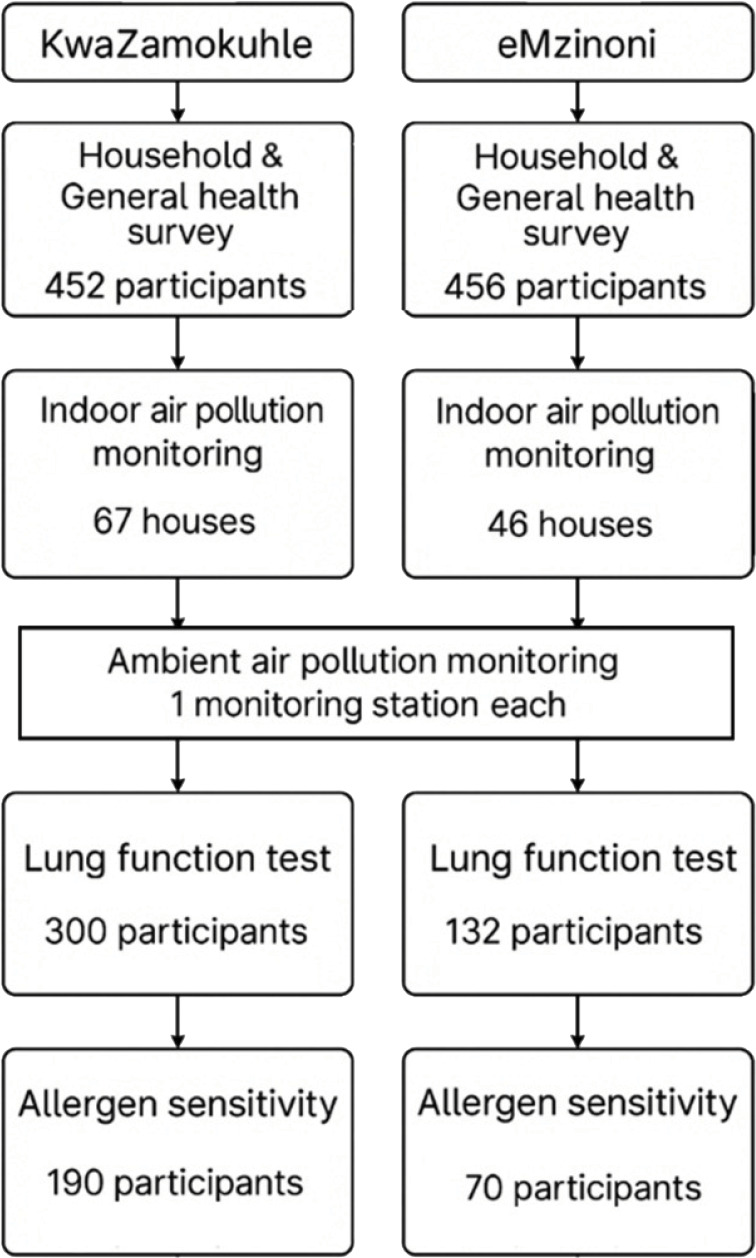
Flowchart of the number of households and participants enrolled in the study.

While we enrolled 1005 participants overall, a total of 908 participants completed both the household questionnaire and general health surveys (Figure 3). Fewer participants agreed to participate in the lung function and allergen sensitivity tests. Indoor air pollution monitoring was conducted in a subsample of participants’ dwellings due to a limited number of instruments.

### Socio-demographics of the participants

All participants were female and over the age of 18 (Table 1). The proportion of participants who completed primary or high school was twice as high in eMzinoni compared to KwaZamokuhle. The employment rates of participants were the same in both communities. Almost a third of all participants stated that their household income was less than ZAR 2000.00/month (USD 106.00). More participants in KwaZamokuhle (*n* = 36) featured below the food poverty line (USD 40 per month) than in eMzinoni (*n* = 11). After adjusting for employment status, monthly income, education and age (with eMzinoni as the reference site), the observed differences between KwaZamokuhle and eMzinoni were found to be significantly different (Table 2).

**Table 1:** Overview of household demographics and characteristics of the participating households in KwaZamokuhle and eMzinoni.

VARIABLE	CHARACTERISTICS OF THE SAMPLE	KWAZAMOKUHLE (*N* = 452) *N* (%)	EMZINONI (N = 456) *N* (%)
**Age of cook (mean and [range])** **Age category**	18–29 years 30–59 years 60+ years	43 [18-91] 113 (25) 269 (60) 70 (15)	48 [18-96] 90 (20) 228 (50) 138 (30)
**Education**	Currently in primary or high school Finished primary school or high school Studying towards a diploma/degree Completed tertiary education Other	11(2) 154 (34) 5 (1) 28 (6) 254 (56)	7 (1) 287 (63) 4 (1) 4 (1) 154 (34)
**Monthly income of household**	< Food poverty line* ZAR 760/month (USD 40/month) < Lower-bound poverty line ZAR 1058/month (USD 56/month) < Upper-bound poverty line ZAR 1558 (USD 83) ZAR 1559–ZAR 2000 (USD 84–107) ZAR 2001–ZAR 5000 (USD 108–267) ZAR 5001–ZAR 10 000 (USD 268–534) ZAR 10 001–ZAR 20 000 (USD 435–1068) ZAR 20 001–ZAR 50 000 (USD 1069–2670) Did not report	36 (8) 24 (5) 22 (5) 56 (12) 77 (17) 19 (4) 24 (5) 10 (2) 184 (41)	11 (2) 22 (5) 17 (4) 107 (23) 70 (15) 14 (3) 3 (1) 0 (0) 212 (46)
**Employment**	Currently employed Currently unemployed	379 (84) 73 (16)	381 (84) 75 (16)
**Food**	Eat less due to money shortage Household runs out of money for food	189 (42) 349 (77)	327 (72) 338 (74)
**Main source of water**	Indoor tap Outdoor tap Outdoor tap away from the dwelling or tank	135 (30) 316 (69) 1 (0.2)	184 (40) 269 (59) 3 (1)
**Water and sanitation**	Toilet is located inside the dwelling	282 (62)	130 (29)
**Waste collection** *If waste not collected*	Waste is collected Waste is buried Burn it Dispose of it in open space Take to municipal dump Wait for next pick-up	451 (99) 0 (0) 1 (0.2) 16 (4) 0 (0) 0 (0)	454 (99) 14 (3) 21 (5) 161 (37) 117 (27) 113 (26)

### Sources of air pollution and fuel use patterns

Despite widespread electricity access for cooking, 55% of participants in KwaZamokuhle and 25% in eMzinoni used coal for cooking. This highlights the ongoing energy insecurity and potential exposure to harmful pollutants. These fuel use patterns reveal key differences between the two communities studied that may compound environmental health risks. A third of participants in KwaZamokuhle (33%) and more households in eMzinoni (41%) reported that they have stoves that made their homes smoky (Table 2).

**Table 2:** Primary fuels for cooking, heating and lighting and main stove types used for cooking in participating households in KwaZamokuhle and eMzinoni.

VARIABLE	KWAZAMOKUHLE (*N* = 452) *N* (%)	EMZINONI (*N* = 456) *N* (%)	χ^2^ VALUE	DF	*p*-VALUE
**Cooking** Electricity Coal LPG Paraffin Wood Animal dung Candle Other	186 (41) 251 (55) 5 (1) 0 (0) 10 (2) 0 (0.0) 0 (0.0) 0 (0.0)	335 (73) 117 (25) 2 (0.4) 1 (0.2) 1 (0.2) 0 (0.0) 0 (0.0) 0 (0.0)	101.0	4	**<0.001**
**Heating** Electricity Coal LPG Paraffin Wood Animal dung Candle Other	12 (2) 405 (89) 6 (1) 0 (0.0) 18 (3) 0 (0.0) 0 (0.0) 11 (2)	151 (33) 298 (65) 4 (0.8) 0 (0.0) 2 (0.4) 0 (0.0) 0 (0.0) 1 (0.2)	156.3	4	**<0.001**
**Lighting** Electricity LPG Other	451 (99) 1 (0.2) 0 (0.0)	453 (99) 1 (0.2) 2 (0.4)	1.9	2	0.370
**Stove type used** Hybrid (electric + LPG) Electric LPG Paraffin Mbaula Cast iron stove Own welded stove Other	4 (0.9) 178 (39) 5 (1) 0 (0) 0 (0) 236 (52) 29 (6) 0 (0.0)	19 (4) 197 (43) 1 (0.2) 0 (0) 1 (0.2) 197 (43) 39 (9) 2 (0.4)	21.3	6	**0.002**
**Stove type** Electric Non-electric	216 (47) 240 (53)	182 (40) 270 (60)	4.6	1	**0.031**
**Stove smoke inside** Yes No	149 (33) 303 (67)	189 (41) 267 (59)	6.99	1	**0.008**

χ^2^value = chi-square value; Df = degrees of freedom; bold font denotes statistical significance for the *p*-values.

Coal was the most commonly used fuel to heat homes. Coal was used for cooking by more than twice as many households in KwaZamokuhle than in eMzinoni and for heating in more households in KwaZamokuhle than in eMzinoni. LPG and wood were used by between 1 and 2% of households as a main cooking fuel.

Electric and LPG stoves were used by slightly more households in eMzinoni while cast iron and welded stoves for solid fuels were used in more households in KwaZamokuhle than in eMzinoni (Table 2). A statistically significant difference was found between the communities for both cooking and heating energy sources. There was also a statistically significant difference in the types of stoves used between the two towns (Table 2). Almost all households used electricity for lighting.

Only 7.5% of individuals in the households smoked. Among these, only half smoked inside the house (*n* = 38 across both communities). The most reported potential source of indoor air pollution was dust followed by mould and pets.

### Indoor and ambient air quality

**Figure 4:**
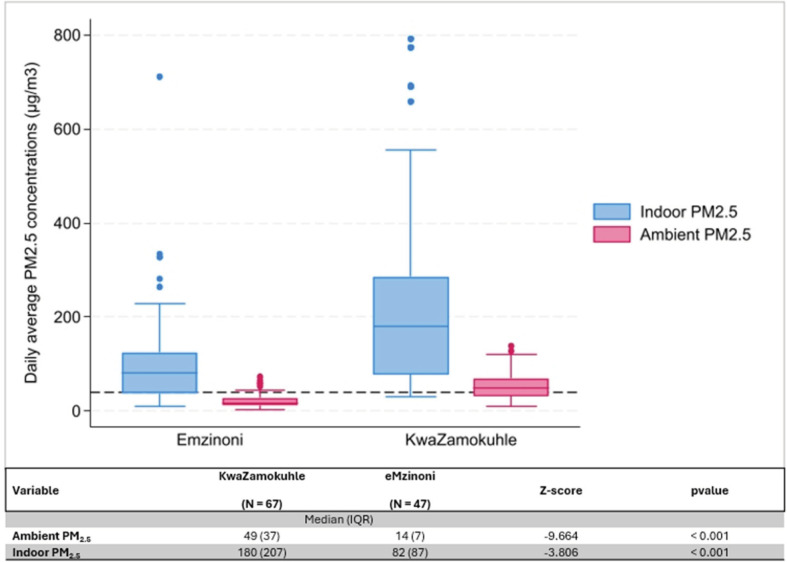
Boxplots of daily average indoor and ambient PM_2‧5_ concentrations (µg/m^3^) in eMzinoni and KwaZamokuhle, with the NAAQS daily limit of 40 µg/m^3^ shown as a dashed line. The nested table compares daily averages between the two sites. Note: IQR = interquartile range.

Indoor and ambient PM_2.5_ levels were more than twice as high in KwaZamokuhle than in eMzinoni (Figure 4). Indoor concentrations showed large interquartile ranges (IQR) (Figure 4). The difference between the average indoor and ambient PM_2.5_ concentrations in the two towns was statistically significant.

### Measured lung function and allergy susceptibility

Participants from KwaZamokuhle had a higher median FVC (2.99 L, IQR 0.52) compared to those from eMzinoni (2.84 L, IQR 0.98). Similarly, the median FEV was higher in KwaZamokuhle (2.59 L, IQR 0.62) than in eMzinoni (2.27 L, IQR 0.93). Medians are reported instead of means due to non-parametric distribution of the data, with interquartile ranges used to reflect variability and reduce the influence of outliers, which is common when reporting data from respiratory diseases [31]. The FEV1/FVC ratios were statistically significantly different between the two towns with participants from eMzinoni having a lower median ratio compared to participants from KwaZamokuhle (Table 3). Participants in both communities showed a non-age-standardized obstructive airway disease prevalence of 9%.

**Table 3:** Comparison of lung function and allergen sensitivity of participants in KwaZamokuhle and eMzinoni.

VARIABLE	KWAZAMOKUHLE (*N* = 300)	EMZINONI (*N* = 132)	*Z* VALUE	*p*-VALUE
**Median Mean FEV1**	2.59 (0.62)	2.27 (0.93)	-3.091	0.002
**Median Mean FVC**	2.99 (0.52)	2.84 (0.98)	-2.673	0.0084
FEV/FVC^a^	0.85 (0.1)	0.82 (0.1)	-3.036	**0.002**
Phadiatop concentration^a^	2.0 (5)	2.9 (11)	-0.822	0.411
Obstructive airways disease^b^	29 (9)	12 (9)	0.035	0.851
Phadiatop interpretation positive^b^	53 (27)	17 (24)	0.339	0.561

^a^Mann–Whitney *U* Test (median IQR); ^b^chi-square (*n* (%)).

The median concentration of allergen-specific IgE antibodies detected in the blood samples of participants in eMzinoni was higher than in KwaZamokuhle, but this was not statistically significant. About one quarter of all participants had positive allergen sensitivity test results (>0.35 kU/L) suggestive of sensitisation to the specific inhalant allergens of the Phadiotop test and the potential to react upon exposure.

**Figure 5:**
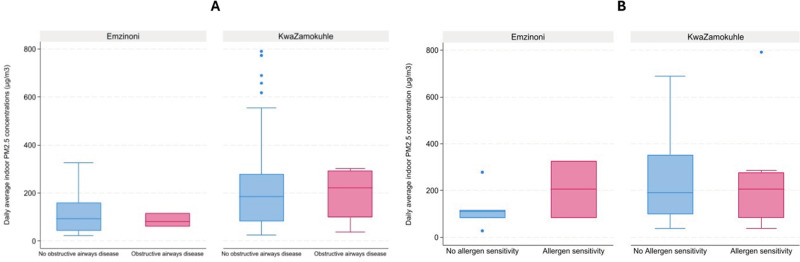
**(A)** Boxplots of obstructive airways disease occurrence at daily average indoor PM_2‧5_ concentrations in selected households in eMzinoni and KwaZamokuhle. Mann–Whitney *U* tests showed no significant differences (eMzinoni: *Z* = 0.00, *p* = 1.00; KwaZamokuhle: *Z* = −0.07, *p* = 0.95). **(B)** Boxplots of allergen sensitivity occurrence at daily average indoor PM_2.5_ concentrations in selected households in eMzinoni and in KwaZamokuhle. Mann–Whitney U test: eMzinoni Z = *−0.78*, *p* = 0.44; KwaZamokuhle *Z* = 0.31, *p* = 0.76).

Analyses showed no statistically significant difference in PM_2.5_ exposure levels between participants with obstructive airways disease and those without the condition (Figure 5A). The same was true for the PM_2.5_ concentrations to which those were exposed who presented with positive and negative allergen sensitivity test results (Figure 5B). Nevertheless, in both communities, the median indoor concentration to which allergen-sensitive individuals were exposed was higher than that to which non-sensitive individuals were exposed, though only marginally so in KwaZamokuhle (Figure 5B). Only in KwaZamokuhle were the indoor PM_2.5_ concentrations higher in those households in which participants who were diagnosed with obstructive airways disease lived.

The median values of FEV1/FVC and Phadiatop concentration were not statistically different across the different fuel uses, stove types used or the presence and absence of stove smoke from the stove in both communities (Tables S2–S5).

While direct associations between fuel use, stove type, and respiratory outcomes were not statistically significant, likely due to limited sample size or unmeasured confounding, the observed trends, particularly in heating fuel use and obstructive airway disease, indicate possible associations. Importantly, ORs > 2 for heating fuel use suggest potentially meaningful health impacts (Table 4).

**Table 4:** Crude and adjusted odds ratio estimates of the effects of various HAP exposure proxy metrics on measured lung function and allergen sensitivity. Results of logistic regression analysis. See notes below Table.

OUTCOMES AND INDEPENDENT VARIABLES	OR (95% CI) AND *P*-VALUE		
	MODEL: BASELINE^a^	MODEL: LIVING CONDITIONS^b^	MODEL: SOCIO-ECONOMIC FACTORS^c^
**Obstructive airways disease**			
**Main cooking fuel** **Electric** **Non-electric**	*Reference* 1.02 (0.54–1.96) *p* = 0.93	*Reference* 0.90 (0.43–2.02) *p* = 0.80	*Reference* 0.82 (0.32 – 2.11) *p* = 0.74
**Main heating fuel** **Electric** **Non-electric**	*Reference* 2.16 (0.50 – 9.29) *p* = 0.30	Reference* 2.28 (0.49 – 10.60) *p* = 0.294	Reference** 1.90 (0.43 – 8.37) *p* = 0.40
**Stove type** **Electric** **Non-electric**	*Reference* 0.87 (0.46 – 1.66) *p* = 0.68	*Reference* 0.91 (0.41 – 2.03) *p* = 0.82	*Reference* 0.90 (0.36 – 2.29) *p* = 0.72
**Stove smoke** **No** **Yes**	*Reference* 0.97 (0.50 – 1.89) *p* = 0.92	*Reference* 0.99 (0.43 – 2.28) *p* = 0.99	*Reference* 1.18 (0.46 – 3.06) *p* = 0.73
**Town** **eMzinoni** **KwaZamokuhle**	*Reference* 1.07 (0.53 – 2.17) *p* = 0.85	*Reference* 1.81 (0.66 – 5.00) *p* = 0.25	*Reference* 0.76 (0.25 – 2.28) *p* = 0.63
***Allergen sensitivity***			
**Main cooking fuel** **Electric** **Non-electric**	*Reference* 1.17 (0.68 – 2.02) *p* = 0.58	*Reference* 1.13 (0.64 – 2.00) *p* = 0.67	*Reference* 1.36 (0.63 – 2.90) *p* = 0.43
**Main heating fuel** **Electric** **Non-electric**	*Reference* 0.78 (0.29 – 2.15) *p* = 0.64	*Reference* 0.60 (0.19 – 1.87) *p* = 0.38	*Reference* 0.86 (0.21 – 3.49) *p* = 0.83
**Stove type** **Electric** **Non-electric**	*Reference* 0.87 (0.50 – 1.52) *p* = 0.62	*Reference* 0.87 (0.50 – 1.54) *p* = 0.64	*Reference* 0.80 (0.38 – 1.70) *p* = 0.56
**Stove smoke** **No** **Yes**	*Reference* 0.70 (0.40 – 1.26) *p* = 0.24	*Reference* 0.76 (0.42 – 1.38) *p* = 0.37	*Reference* 0.75 (0.33 – 1.69) *p* = 0.48
**Town** **eMzinoni** **KwaZamokuhle**	*Reference* 1.21 (0.0.64 – 2.27) *p* = 0.56	*Reference* 1.27 (0.67 – 2.41) *p* = 0.46	*Reference* 0.71 (0.30 – 1.71) *p* = 0.45

*Excluded time lived in community due to collinearity issues.

**Excluded monthly income, due to collinearity issues.

^a^Unadjusted bivariate model.

^b^Adjusted for time lived in community and whether or not the home has a ceiling.

^c^Adjusted for employment status, monthly income, education and age.

Notably, the ORs for non-electric heating fuel use were consistently above ‘2’ across models, suggesting a possible elevated risk for obstructive airway disease, even though statistical significance was not reached. Wide confidence intervals underscore uncertainty but also reflect real-world variability that needs to be captured in larger or more targeted studies. Notably, the ORs for the other exposure variables fluctuated around ‘1’ for the ‘baseline’ and the ‘living conditions’ models. Overall, the results suggest positive associations between solid fuels used for heating and decreased respiratory health, which could compound with time.

## Discussion

This study investigated sources of indoor air pollution, solid fuel use patterns and their association with respiratory health outcomes in two South African communities based in a high air pollution priority area in Mpumalanga. Both communities displayed significantly higher indoor air pollution compared to ambient air pollution. Since disparities between indoor and ambient pollutant levels would not exist if cleaner fuels or electricity were used, it is evident that the usage of solid fuels was a major influence on household air pollution in this population.

The measured PM exposure readings in both communities support these findings of unacceptably higher PM_2.5_ concentrations in the indoor than in the ambient environment in low-income communities in Mpumalanga, with chronic and acute values reaching levels that exceed the local NAAQS [32–34]. In several studies, the use of coal for heating and somewhat less for cooking causes higher levels of indoor pollutants [35–38]. In this study, there was a more frequent use of coal for cooking in KwaZamokuhle (55%) than in eMzinoni (25%). There are numerous determinants of fuel use patterns in low-income communities and these are highly contextual [39]. A previous source apportionment study in KwaZamokuhle showed that coal combustion contributed substantially to PM_2.5_ levels in winter (up to 52%) and meaningfully to PM_2.5_ levels in summer (38%) [40, 41]. In KwaZamokuhle and eMzinoni, geographical location, access to electricity and fuel choice at a household level differed. This is possibly a result of behavioural elements, cultural practices, or even local economic factors [39]. While no specific studies exist for eMzinoni, wood, biomass, coal burning, soil and road dust, secondary aerosols and industry have been identified as major PM contributors in similar low-income areas [40–43]. In eMzinoni, 5% of households burn waste when uncollected, a common practice in South African low-income households [44].

KwaZamokuhle exhibited more than twice the indoor and ambient PM_2‧5_ levels of eMzinoni, accompanied by a greater reliance on coal for cooking and heating. These elevated pollution levels are well above the WHO reference standards for health. The fact that KwaZamokuhle showed a lower socioeconomic status compared to eMzinoni raises environmental justice concerns and underscores the need for localised attention to the more disadvantaged communities, particularly those below the food poverty index. Studies in other low-income countries show that low-income communities face compounded exposures due to low socioeconomic status leading to greater numbers of premature deaths attributable to PM_2‧5_ exposure [45] and are disproportionately affected as there is very little that can be done to limit pollutant exposure [46].

The KwaZamokuhle–eMzinoni disparity exemplifies global patterns where coal-dependent communities face environmental injustice through disproportionate PM_2‧5_ exposure. Effective interventions require multi-pronged approaches combining improved stoves, better ventilation, fuel switching and community engagement. The amplification of health risks through combined indoor and ambient exposures necessitates addressing both sources simultaneously. Studies from South Africa show that the availability of low-cost coal within the area makes it the fuel of choice for heating and cooking purposes [47]. This results in residents being exposed to high concentrations of air pollutants [47]. Given KwaZamokuhle’s high indoor and ambient PM_2.5_ concentrations and high coal usage for heating and cooking, coal remains a large contributor to PM_2.5_ levels.

Given that women disproportionately bear the burden of household cooking tasks, this study brings needed attention to the gendered dimensions of environmental health risk. Studies show that women’s routine exposure to smoke from solid fuels places them at heightened risk of chronic respiratory conditions, and the potential for cumulative, lifelong impacts is a serious concern for public health and gender equity [48]. The introduction of cooking with electricity has improved the health outcomes of women who would otherwise have direct and frequent exposure to solid fuel emissions. However, there is still a significant reliance on solid fuels due to it being a cheaper source of energy in low-income settings. Since access to electricity for cooking and lighting is higher, the continued reliance on coal for heating, particularly in KwaZamokuhle, reflects seasonal vulnerabilities and a gap in thermal energy security. This study suggests that these practices are likely influenced by affordability, cultural norms and household infrastructure, underscoring the need for holistic and accessible energy transition strategies.

Although the average lung function in both communities remained within the normal range, indicating no severe impairment, residents of eMzinoni exhibited significantly lower lung function compared to those in KwaZamokuhle. However, without data from an area with fresher air for direct comparison, it is not possible to determine how much lung function in either community deviates from that of populations living away from industrial activities with better air quality. Moreover, this cross-sectional data does not rule out the effects of chronic exposure to HAP. The percentage of participants who had detectable levels of specific IgE antibodies in response to the inhalant allergens was consistent with the regional prevalence. Respiratory allergies affect 20–30% of the population of Southern Africa, significantly impacting morbidity, employment absenteeism, loss of quality of life and mortality [49, 50]. Many households reported the presence of risk factors for respiratory allergies. Local research shows an association between exposure to indoor bioaerosols and allergens and airway inflammation and asthma [51, 52]. High PM_2.5_ levels might amplify allergic reactions as PM can boost immune responses to allergens causing inflammation [53, 54, 26]

The higher incidence of coal use in KwaZamokuhle than in eMzinoni is associated with higher indoor and ambient PM concentrations, but no association between HAP exposure and obstructive airways disease or allergen sensitivity was identified.

Although the findings of associations between the key outcomes of obstructive airways disease and sensitisation were not statistically significant, the direction of effect implied that pollution could contribute adversely to these outcomes in this sample. A systematic review study focusing on low-income communities in the Global South illustrated mixed findings when assessing adult HAP exposure’s role in the development of obstructive airways disease, with some studies showing low prevalence of obstructive airways disease and no association with biomass smoke exposure and others showing a clear association between the two [55]. Many confounding factors make it difficult to isolate the effects of HAP exposure on respiratory health [56].

### Strengths, limitations and recommendations for further research

A key strength of this study is the rare combination of measured respiratory health and household air pollution (HAP) exposure data in these low-income communities in Mpumalanga, which provides an important evidence base for understanding local health risks. Data were collected directly from participants using standardised protocols, ensuring internal consistency. The study also adjusted multiple regression models for relevant confounders, strengthening the validity of the effect estimates. Despite the lack of statistically significant associations, the presence of effects in the expected direction suggests that exposure-related adverse effects may be occurring in this sample. This study also provides valuable contextual insights that can inform the design and implementation of larger, more robust investigations in similar settings.

Several factors may have contributed to the absence of statistically significant associations between the exposure variables (fuel and stove types) and lung function-related outcomes. Exposure misclassification is an important consideration and due to limited resources, only a sub-sample of homes was monitored for PM, and these measurements were based on single samples. Evidence from previous research [19] indicates considerable inter- and intra-household variability in exposure, meaning our single-day sampling approach may not have fully captured participants’ true exposure levels. The relatively small sample size is another limitation; the targeted sample size was not reached due to study challenges, and because the effect sizes for lung function are likely to be small in cross-sectional studies, larger samples are typically required to detect significant effects.

Data collection was further challenged by external factors, including the COVID-19 pandemic, which restricted the collection of measured health data. Community unrest, the absence of household members during scheduled visits, and inaccessible roads due to heavy rainfall also contributed to the reduced sample size. Owing to resource constraints, one daily average measurement per dwelling was used to determine indoor PM exposure, although multi-day (≥48-hour) measurements are generally preferred. Ambient PM_2‧5_ concentrations were measured at centrally located monitoring stations, and future studies should incorporate multi-day and continuous monitoring to improve exposure assessment.

Future research should employ larger sample sizes and longitudinal designs to better elucidate mechanistic associations between HAP exposure and respiratory health outcomes. This should include multi-day and continuous exposure data, early-life exposures and post-bronchodilator spirometry. Further investigation into the role of allergen exposure and sensitisation, particularly in relation to increased morbidity among participants with obstructive airways disease, is also warranted.

## Conclusions

Household fuel use, HAP and ambient air pollution, as well as air pollution-related health outcomes, were compared across two low-income communities in Mpumalanga, South Africa. The community with a higher prevalence of coal use had substantially higher indoor and ambient PM_2.5_ concentrations. To the best of our knowledge, this is one of the first studies to conduct comprehensive assessments of respiratory health outcomes related to HAP and ambient air pollution exposure in Mpumalanga. Air pollution concentrations inside houses were high and therefore pose a grave risk to the health and well-being of the inhabitants. Urgent attention is required to address HAP in these areas and areas with similar cooking and heating patterns.

## Declaration

All authors had access to the data and had a role in writing the manuscript.

## Additional Files

The additional files for this article can be found as follows:

Supplementary Table 1. Extract of questions from the household and general health survey used for this study.

Supplementary Table 2. Overview of chi-square results for KwaZamokuhle and eMzinoni presenting fuel use patterns, stove use and the presence and absence of smoke in the dwelling for people with and without COPD, based on their lung function test results.

Supplementary Table 3. Overview of Kruskal–Wallis test results for KwaZamokuhle and eMzinoni presenting fuel use patterns, stove use and the presence and absence of smoke in the dwelling in the context of FEV/FVC results from the conducted lung function tests.

Supplementary Table 4. Overview of chi-square test results for KwaZamokuhle and eMzinoni presenting fuel use patterns, stove use and the presence and absence of smoke in the dwelling in the context of allergy sensitivity results from the conducted Phadiotop tests.

Supplementary Table 5. Overview of Kruskal–Wallis test results for KwaZamokuhle and eMzinoni presenting fuel use patterns, stove use and the presence and absence of smoke in the dwelling in the context of allergy sensitivity results from the conducted Phadiotop tests.
