# Supplementary material for: Impact of Solid Fuel Use on Household Air Pollution and Respiratory Health in Two Low-Income Communities in Mpumalanga, South Africa
Source: Ann Glob Health. 2025 Oct 8;91(1):70. doi: 10.5334/aogh.4923 (PMC12513343; doi:10.5334/aogh.4923)
Supplement: Supplementary Table 1. — Extract of questions from the household and general health survey used for this study. [file agh-91-1-4923-s1.pdf]

Supplementary material

Table S1 Extract of questions from the household and general health survey used for this study.

**Question**	**Response options**
Demographics and socio-economic status	
What is your age (in years)?	[Number of years]
What is the highest qualification for PERSON NAME?	• Currently in primary school;<br>• Finished primary school;<br>• Currently in high school;<br>• Finished high school;<br>• Currently studying towards a tertiary qualification degree diploma;<br>• Completed tertiary qualification degree diploma;<br>• Other.
What is the total monthly income (in Rand) for this household? (Fieldworker: you can skip this question if the respondent is unwilling to disclose their income.)	[Amount in ZAR]
Which of the following best describes the situation of PERSON NAME?	• Retired person pensioner;<br>• Unable to work due to disability;<br>• Unemployed, not wishing to work;<br>• Unemployed looked for work in past three months;<br>• Unemployed, not looking for work but will accept work;<br>• Housewife homemaker;<br>• Self-employed, e.g., selling goods, hair salon etc.;<br>• Part-time employed, including seasonal work;<br>• Paid full-time employment;

	• School pupil full-time student;<br>• School going age not going to school;<br>• Too young for school;<br>• Is in a crèche daycare centre during the day.
Do you ever eat less than you should because there is not enough money for food?	• Yes<br>• No
Does this household ever run out of money to buy food?	• Yes<br>• No
Housing conditions/ household characteristics	
Where is your toilet located?	• Inside the house<br>• Outside the house
What is the main source of water for this household?	• Indoor tap;<br>• Outdoor tap at the house;<br>• Outdoor tap (away from the house) within 200m of the house;<br>• Outdoor tap (away from the house) more than 200m from the house;<br>• Water tanks such as JoJo tank;<br>• Borehole;<br>• Spring point at which water flows from the ground to the earth's surface;<br>• Rainwater collected and stored;<br>• Dam pool;<br>• River stream;<br>• Water vendor.
Is your household waste collected by the municipality or another agency?	Yes<br>No
What do you do with your waste when it is not collected?	• Bury it;<br>• Burn it;

	• Dispose of it in nearby open spaces;<br>• Take it to the municipal waste dump site;<br>• Other.
Does your stove make the house smoky inside?	• Yes<br>• No
Does the house have a ceiling?	• Yes, some rooms<br>• Yes, all rooms<br>• No
Main energy use	
Which fuel do you use most of the time to cook? (Tick one option only)	• Electricity;<br>• Gas;<br>• Paraffin;<br>• Wood;<br>• Coal;<br>• Other.
Which fuel do you use most of the time to heat your house? (Tick one option only)	• Electricity;<br>• Gas;<br>• Paraffin;<br>• Wood;<br>• Coal;<br>• Other.
Which fuel do you use most of the time for lighting? (Tick one option only)	• Electricity;<br>• Gas;<br>• Paraffin;<br>• Other.
Health	
Acute illness:<br>During the past two weeks, has a doctor or nurse diagnosed PERSON NAME with one or more of the following illnesses?	• Stroke;<br>• Heart attack;<br>• Pneumonia;<br>• Bronchitis non-chronic;

	• Meningitis;<br>• Vomiting;<br>• Diarrhoea;<br>• None of the above.
Chronic illness:<br><br>Has a doctor or nurse ever diagnosed PERSON NAME with one or more of the following illnesses? You do not have to disclose this information if you feel uncomfortable doing so.	• High blood pressure/ hypertension;<br>• Diabetes/ High blood sugar;<br>• Arthritis;<br>• Tuberculosis;<br>• Heart failure;<br>• Asthma;<br>• Chronic lung disease/ Bronchitis;<br>• Headaches;<br>• Cancer;<br>• HIV/ AIDS;<br>• None of the above.
Does PERSON NAME smoke?	• Yes<br>• No
Does PERSON NAME smoke inside the house?	• Yes<br>• No

Note: The analysis did not include data on household smoking, as only 7.5% of individuals in the households smoked. Among these, only half smoked inside the house (n = 38 across both communities), and no discernible effect was established.
