# Supplementary material for: Impact of Solid Fuel Use on Household Air Pollution and Respiratory Health in Two Low-Income Communities in Mpumalanga, South Africa
Source: Ann Glob Health. 2025 Oct 8;91(1):70. doi: 10.5334/aogh.4923 (PMC12513343; doi:10.5334/aogh.4923)
Supplement: Supplementary Table 2. — Overview of chi-square results for KwaZamokuhle and eMzinoni presenting fuel use patterns, stove use and the presence and absence of smoke in the dwelling for people with and without COPD, based on their lung function test results. [file agh-91-1-4923-s2.pdf]

Supplementary material

Table S2 Overview of chi-square results for KwaZamokuhle and eMzinoni presenting fuel use patterns, stove use and the presence and absence of smoke in the dwelling for people with and without COPD, based on their lung function test results.

		**KwaZamokuhle**				**eMzinoni**			
		**N=300**				**N=132**			
**Variable**	**Variable Category**	**COPD** *Yes*	**COPD** *No*	χ^2	**p-value**	**COPD** *Yes*	**COPD** *No*	χ^2	**p-value**
**Main cooking fuel**	*Electricity*	14	114	0.8386	0.840	7	89	1.6189	0.445
	LPG	0	2			-	-		
	Wood	1	6			0	1		
	Coal	14	149			5	30		
	Total	***29***	***271***			***12***	***120***		
		**KwaZamokuhle**				**eMzinoni**			
		**N=300**				**N=132**			
**Variable**	**Variable Category**	**COPD** *Yes*	**COPD** *No*	χ^2	**P-value**	**COPD** *Yes*	**COPD** *No*	χ^2	**P-value**
**Main heating fuel**	*Electricity*	0	5	1.3398	0.855	2	34	1.2966	0.862
	LPG	0	4			0	2		
	Wood	1	11			0	1		
	Coal	27	246			10	82		
	Other	1	5			0	1		
	Total	***29***	***271***			***12***	***120***		
		**KwaZamokuhle**				**eMzinoni**			
		**N=300**				**N=132**			
**Variable**	**Variable Category**	**COPD** *Yes*	**COPD** *No*	χ^2	**P-value**	**COPD** *Yes*	**COPD** *No*	χ^2	**P-value**
**Main lighting fuel**	*Electricity*	29	270	0.107	0.743	12	119	0.101	0.751
	LPG	0	1			-	-		
	Other	-	-			0	1		
	Total	***29***	***271***			***12***	***120***		

		KwaZamokuhle N=300				eMzinoni N=132			
**Variable**	**Variable Category**	**COPD** *Yes*	**COPD** *No*	**X^2**	**P-value**	**COPD** *Yes*	**COPD** *No*	**X^2**	**P-value**
**Stove use**	*Hybrid (electric + LPG)*	1	3	3.6192	0.460	0	5	1.371	0.927
	Electric	13	107			5	52		
	LPG	0	2			-	-		
	Paraffin	-	-			-	-		
	Mbaula	-	-			0	1		
	Cast iron stove	15	139			7	57		
	Own welded stove	0	20			0	4		
	Other	-	-			0	1		
	Total	***29***	***271***			***12***	***120***		

		KwaZamokuhle N=300				eMzinoni N=132			
**Variable**	**Variable Category**	**COPD** *Yes*	**COPD** *No*	**X^2**	**P-value**	**COPD** *Yes*	**COPD** *No*	**X^2**	**P-value**
**Stove smoke**	*Yes*	9	89	0.040	0.844	6	57	0.027	0.869
	No	20	182			6	63		
	Total	***29***	***271***			***12***	***120***		
