# Supplementary material for: Impact of Solid Fuel Use on Household Air Pollution and Respiratory Health in Two Low-Income Communities in Mpumalanga, South Africa
Source: Ann Glob Health. 2025 Oct 8;91(1):70. doi: 10.5334/aogh.4923 (PMC12513343; doi:10.5334/aogh.4923)
Supplement: Supplementary Table 3. — Overview of Kruskal–Wallis test results for KwaZamokuhle and eMzinoni presenting fuel use patterns, stove use and the presence and absence of smoke in the dwelling in the context of FEV/FVC results from the conducted lung function tests. [file agh-91-1-4923-s3.pdf]

**Supplementary material**

**Table S3** Overview of Kruskal-Wallis test results for KwaZamokuhle and eMzinoni presenting fuel use patterns, stove use and the presence and absence of smoke in the dwelling in the context of FEV/FVC results from the conducted lung function tests.

| | | **KwaZamokuhle** | | | **eMzinoni** | | |
|---|---|---|---|---|---|---|---|
| | | **N=300** | | | **N=132** | | |
| **Variable** | **Variable Category** | **FEV/FVC**<br>**n** (median IQR) | H statistic | **P-value** | **FEV/FVC** | H statistic | **P-value** |
| **Main cooking fuel** | *Electricity* | n = 128<br>0.85 (0.12) | 0.431 | 0.9338 | n = 96<br>0.82 (0.9) | 3.616 | 0.164 |
| | *LPG* | n = 2<br>0.84 (0.03) | | | - | | |
| | *Wood* | n = 7<br>0.82 (0.17) | | | n = 1<br>0.93 (0) | | |
| | *Coal* | n = 163<br>0.86 (0.12) | | | n = 35<br>0.8 (0.11) | | |
| | ***Total*** | ***300*** | | | ***132*** | | |
| | | **KwaZamokuhle** | | | **eMzinoni** | | |
| | | **N=300** | | | **N=132** | | |
| **Variable** | **Variable Category** | **FEV/FVC Median (IQR)** | H statistic | **P-value** | **FEV/FVC Median (IQR)** | H statistic | **P-value** |
| **Main heating fuel** | *Electricity* | n = 5<br>0.83 (0.04) | 2.207 | 0.6978 | n = 36<br>0.83 (0.06) | 2.698 | 0.6969 |
| | *LPG* | n = 4<br>0.86 (0.8) | | | n = 2<br>0.80 (0.05) | | |
| | *Wood* | n = 12<br>0.89 (0.10) | | | n = 1<br>0.78 (0) | | |
| | *Coal* | n = 273<br>0.85 (0.13) | | | n = 92<br>0.82 (0.12) | | |
| | *Other* | n = 6<br>0.85 (0.04) | | | n = 1<br>0.92 (0) | | |

| | | | | | | | |
|---|---|---|---|---|---|---|---|
| | ***Total*** | ***300*** | | | ***132*** | | |
| | | **KwaZamokuhle** | | | **eMzinoni** | | |
| | | **N=300** | | | **N=132** | | |
| **Variable** | **Variable Category** | **FEV/FVC Median (IQR)** | H statistic | **P-value** | **FEV/FVC Median (IQR)** | H statistic | **P-value** |
| **Main lighting fuel** | *Electricity* | n = 299<br>0.85 (0.13) | 0.488 | 0.485 | n = 131<br>0.82 (0.1) | 0.031 | 0.885 |
| | *LPG* | n = 1<br>0.89 (0) | | | - | | |
| | *Other* | - | | | n = 1<br>0.81 (0) | | |
| | ***Total*** | ***300*** | | | ***132*** | | |

| | | | | | | | |
|---|---|---|---|---|---|---|---|
| | | **KwaZamokuhle** | | | **eMzinoni** | | |
| | | **N=300** | | | **N=132** | | |
| **Variable** | **Variable Category** | **FEV/FVC Median (IQR)** | H statistic | **P-value** | **FEV/FVC Median (IQR)** | H statistic | **P-value** |
| **Stove use** | *Hybrid (electric + LPG)* | n = 4<br>0.84 (0.11) | 1.297 | 0.862 | n = 5<br>0.80 (0.06) | 3.864 | 0.569 |
| | *Electric* | n = 120<br>0.85 (0.13) | | | n = 57<br>0.82 (0.09) | | |
| | *LPG* | n = 2<br>0.84 (0.03) | | | - | | |
| | *Paraffin* | - | | | - | | |
| | *Mbaula* | - | | | n = 1<br>0.75 (0) | | |
| | *Cast iron stove* | n = 154<br>0.85 (0.12) | | | n = 64<br>0.82 (0.10) | | |
| | *Own welded stove* | n = 20<br>0.87 (0.12) | | | n = 4<br>0.84 (0.4) | | |

| | | | | | | | |
|---|---|---|---|---|---|---|---|
| | *Other* | - | | | n = 1<br>0.92 (0) | | |
| | ***Total*** | ***300*** | | | ***132*** | | |
| | | **KwaZamokuhle** | | | **eMzinoni** | | |
| | | **N=300** | | | **N=132** | | |
| **Variable** | **Variable Category** | **FEV/FVC Median (IQR)** | H statistic | **P-value** | **FEV/FVC Median (IQR)** | H statistic | **P-value** |
| **Stove smoke** | *Yes* | n = 98<br>0.85 (0.12) | 0.231 | 0.631 | n = 63<br>0.82 (0.09) | 0.277 | 0.599 |
| | *No* | n = 202<br>0.85 (0.12) | | | n = 69<br>0.82 (0.11) | | |
| | ***Total*** | 300 | | | 132 | | |
