# Supplementary material for: Impact of Solid Fuel Use on Household Air Pollution and Respiratory Health in Two Low-Income Communities in Mpumalanga, South Africa
Source: Ann Glob Health. 2025 Oct 8;91(1):70. doi: 10.5334/aogh.4923 (PMC12513343; doi:10.5334/aogh.4923)
Supplement: Supplementary Table 4. — Overview of chi-square test results for KwaZamokuhle and eMzinoni presenting fuel use patterns, stove use and the presence and absence of smoke in the dwelling in the context of allergy sensitivity results from the conducted Phadiotop tests. [file agh-91-1-4923-s4.pdf]

Supplementary material

Table S4 Overview of Chi-square test results for KwaZamokuhle and eMzinoni presenting fuel use patterns, stove use and the presence and absence of smoke in the dwelling in the context of allergy sensitivity results from the conducted Phadiotop tests.

		**KwaZamokuhle**				**eMzinoni**			
		**N=190**				**N=70**			
**Variable**	**Variable Category**	**Allergen Sensitivity**		**χ2**	**P-value**	**Allergen Sensitivity**		**χ2**	**P-value**
		Yes	*No*			*Yes*	*No*		
**Main cooking fuel**	*Electricity*	22	60	3.162	0.367	11	37	0.579	0.748
	LPG	1	1			-	-		
	Wood	3	2			0	1		
	Coal	27	74			6	15		
	Total	***53***	***137***			***17***	***53***		
		**KwaZamokuhle**				**eMzinoni**			
		**N=190**				**N=70**			
**Variable**	**Variable Category**	**Allergen Sensitivity**		**χ2**	**P-value**	**Allergen Sensitivity**		**χ2**	**P-value**
		Yes	*No*			*Yes*	*No*		
**Main heating fuel**	*Electricity*	1	1	1.057	0.901	5	12	1.694	0.792
	LPG	1	3			1	1		
	Wood	2	9			0	1		
	Coal	48	121			11	38		
	Other	1	3			0	1		
	Total	***53***	***137***			***17***	***53***		
		**KwaZamokuhle**				**eMzinoni**			
		**N=190**				**N=70**			
**Variable**	**Variable Category**	**Allergen Sensitivity**		**χ2**	**P-value**	**Allergen Sensitivity**		**χ2**	**P-value**
		Yes	*No*			*Yes*	*No*		
**Main lighting fuel**	*Electricity*	53	136	0.389	0.533	17	52	0.325	0.568
	LPG	0	1			-	-		
	Other	-	-			0	1		
	Total	***53***	***137***			***17***	***53***		

		KwaZamokuhle N=190				eMzinoni N=70			
**Variable**	**Variable Category**	**Allergen Sensitivity**		**χ2**	**P-value**	**Allergen Sensitivity**		**χ2**	**P-value**
		Yes	*No*			*Yes*	*No*		
**Stove use**	*Hybrid (electric + LPG)*	2	1	3.168	0.530	0	1	0.835	0.934
	Electric	22	53			6	20		
	LPG	1	1			-	-		
	Paraffin	-	-			-	-		
	Mbaula	-	-			0	1		
	Cast iron stove	24	69			10	29		
	Own welded stove	4	13			1	2		
	Other	-	-			-	-		
	Total	***53***	***137***			***17***			

		KwaZamokuhle N=190				eMzinoni N=70			
**Variable**	**Variable Category**	**Allergen Sensitivity**		**χ2**	**P-value**	**Allergen Sensitivity**		**χ2**	**P-value**
		Yes	*No*			*Yes*	*No*		
**Stove smoke**	*Yes*	14	50	1.739	0.187	8	25	0.0001	0.994
	No	39	87			9	28		
	Total	***53***	***137***			***17***	***53***		
