# Supplementary material for: Impact of Solid Fuel Use on Household Air Pollution and Respiratory Health in Two Low-Income Communities in Mpumalanga, South Africa
Source: Ann Glob Health. 2025 Oct 8;91(1):70. doi: 10.5334/aogh.4923 (PMC12513343; doi:10.5334/aogh.4923)
Supplement: Supplementary Table 5. — Overview of Kruskal–Wallis test results for KwaZamokuhle and eMzinoni presenting fuel use patterns, stove use and the presence and absence of smoke in the dwelling in the context of allergy sensitivity results from the conducted Phadiotop tests. [file agh-91-1-4923-s5.pdf]

Supplementary material

Table S5 Overview of Kruskal-Wallis test results for KwaZamokuhle and eMzinoni presenting fuel use patterns, stove use and the presence and absence of smoke in the dwelling in the context of allergy sensitivity results from the conducted Phadiotop tests.

		**KwaZamokuhle**			**eMzinoni**		
		**N=190**			**N=70**		
**Variable**	**Variable Category**	Phadiatop concentration **Median (IQR)**	H statistic	**P-value**	Phadiatop concentration **Median (IQR)**	H statistic	**P-value**
**Main cooking fuel**	*Electricity*	n = 82<br>0.12 (0.30)	3.238	0.356	n = 48<br>0.09 (0.26)	2.380	0.3043
	LPG	n = 2<br>0.42 (0.50)			-		
	Wood	n = 5<br>0.70 (1.77)			n = 1<br>0.02 (0.00)		
	Coal	n = 101<br>0.09 (0.48)			n = 21<br>1.00 (0.43)		
	Total	***190***			***70***		
		**KwaZamokuhle**			**eMzinoni**		
		**N=190**			**N=70**		
**Variable**	**Variable Category**	Phadiotop concentration **Median (IQR)**	H statistic	**P-value**	Phadiatop concentration **Median (IQR)**	H statistic	**P-value**
**Main heating fuel**	*Electricity*	n = 2<br>0.25 (0.31)	2.933	0.569	n = 17<br>0.10 (0.36)	5.478	0.242
	LPG	n = 4<br>0.06 (0.40)			n = 2<br>0.95 (1.64)		
	Wood	n = 11<br>0.08 (0.23)			n = 1<br>0.04 (0.00)		
	Coal	n = 169			n = 49		

		0.10 (0.59)			0.08 (0.26)		
	Other	n = 4<br>0.30 (1.35)			n = 1<br>0.02 (0.00)		
	Total	***190***			***70***		
		**KwaZamokuhle**			**eMzinoni**		
		**N=190**			**N=70**		
**Variable**	**Variable Category**	Phadiatop concentration **Median (IQR)**	H statistic	**P-value**	Phadiatop concentration **Median (IQR)**	H statistic	**P-value**
**Main lighting fuel**	*Electricity*	n = 189<br>0.10 (0.49)	1.676	0.196	n = 69<br>0.08 (0.26)	0.088	0.767
	LPG	n = 1<br>0.04 (0.00)			-		
	Other	-			n = 1<br>0.11 (0.00)		
	Total	***190***			***70***		
		**KwaZamokuhle**			**eMzinoni**		
		**N=190**			**N=70**		
**Variable**	**Variable Category**	Phadiatop concentration **Median (IQR)**	H statistic	**P-value**	Phadiatop concentration **Median (IQR)**	H statistic	**P-value**
**Stove use**	*Hybrid (electric + LPG)*	n = 3<br>5.56 (6.80)	5.184	0.2690	n = 1<br>0.07 (0.00)	0.484	0.975
	Electric	n = 75<br>0.14 (0.48)			n = 26<br>0.11 (0.26)		
	LPG	n = 2<br>0.42 (0.49)			-		
	Paraffin	-			-		
	Mbaula	-			n = 1<br>0.08 (0.00)		
	Cast iron stove	n = 93			n = 39		

		0.08 (0.35)			0.07 (0.43)		
	Own welded stove	n = 17<br>0.09 (0.14)			n = 3<br>0.11 (1.36)		
	Other	-			-		
	Total	***190***			***70***		
		**KwaZamokuhle**			**eMzinoni**		
		**N=190**			**N=70**		
**Variable**	**Variable Category**	Phadiatop concentration **Median (IQR)**	H statistic	**P-value**	Phadiatop concentration **Median (IQR)**	H statistic	**P-value**
**Stove smoke**	*Yes*	n = 64<br>0.8 (0.21)	2.226	0.136	n = 33<br>0.08 (0.26)	0.035	0.851
	No	n = 126<br>0.12 (0.6)			n = 37<br>0.10 (0.20)		
	Total	190			70		
